# Hereditary Hemorrhagic Telangiectasia: Success of the Osler Calendar for Documentation of Treatment and Course of Disease

**DOI:** 10.3390/jcm10204720

**Published:** 2021-10-14

**Authors:** Caroline T. Seebauer, Viola Freigang, Franziska E. Schwan, René Fischer, Christopher Bohr, Thomas S. Kühnel, Kornelia E. C. Andorfer

**Affiliations:** 1 Department of Otorhinolaryngology, Regensburg University Medical Center, Franz-Josef-Strauß-Allee 11, 93053 Regensburg, Germany; franziska.schwan@ukr.de (F.E.S.); rene.fischer@ukr.de (R.F.); christopher.bohr@ukr.de (C.B.); thomas.kuehnel@ukr.de (T.S.K.); kornelia.andorfer@ukr.de (K.E.C.A.); 2 Department of Trauma, Regensburg University Medical Center, Franz-Josef-Strauß-Allee 11, 93053 Regensburg, Germany; viola.freigang@ukr.de

**Keywords:** hereditary hemorrhagic telangiectasia, Morbus Osler, Rendu-Osler-Weber syndrome, Osler Calendar, orphan disease, epistaxis, arteriovenous malformations, organ manifestation, screening, laser therapy

## Abstract

Hereditary hemorrhagic telangiectasia (HHT; Rendu-Osler-Weber syndrome) affects the capillary and larger vessels, leading to arteriovenous shunts. Epistaxis is the main symptom impairing quality of life. The aim of the Osler Calendar is to offer information about the extent of the systemic disease and the current state of treatment. A care plan with information on the rare disease and self-treatment of epistaxis was created. Organ examinations and ongoing treatments were recorded. A questionnaire documents the treatment success, including patient satisfaction, frequency of hemorrhage and hemoglobin levels. The patients using the Osler Calendar for at least one year (*n* = 54) were surveyed. Eighty-five percent of patients (*n* = 46) used the calendar to gain information about HHT. Seventy-two percent (*n* = 39) used the Osler Calendar for instructions on the self-treatment of nosebleeds. The calendar increased patients’ understanding for the need for organ screenings from 48% (*n* = 26) to 81% (*n* = 44). Seventy-nine percent (*n* = 43) of patients confirmed that the Osler Calendar documented their therapeutic process either well or very well. Fifty-two percent (*n* = 28) saw an improvement in the therapeutic process due to the documentation. The Osler Calendar records the individual intensity of the disease and facilitates the communication between attending physicians. It is a tool for specialists to review treatment strategies. Furthermore, the calendar enhances patients’ comprehension of their condition.

## 1. Introduction

Hereditary hemorrhagic telangiectasia (HHT; Rendu-Osler-Weber syndrome) is an autosomal, dominant, inherited disorder affecting the capillary and larger vessels in all organs in the human body. The occurring lesions range from very small microvascular dilatations to arteriovenous shunts with a diameter of several centimeters. These vascular malformations can be predominantly found in the nasal mucosa, intestine, lung, liver and the central nervous system.

HHT occurs with a prevalence of between 1:5000 and 1:8000 [1]. Thus, HHT belongs to a category of orphan diseases and should be treated in specialized centers [2]. However, in everyday life patients are in contact with healthcare providers with limited prior exposure or training on this specific disease [3]. A diagnosis is based on clinical symptoms, referred to as the Curaçao criteria. These criteria comprise epistaxis, visceral lesions, a positive family history and telangiectasias involving the perioral region, tongue, oral mucosa and fingers [2]. If at least three of these four criteria are fulfilled, the diagnosis can be regarded as definite. Where only two criteria are satisfied, HHT is possible or suspected [4]. Without genetical testing a suspected diagnosis cannot be discounted as further symptoms may occur over time. Epistaxis is the main presenting symptom and almost every patient diagnosed with HHT is affected by it. The intensity of this symptom varies from mild, scarce bleeding to life-threatening epistaxis requiring nasal packing, hospitalization, and frequent blood transfusion, thus limiting the patient’s quality of life [5]. The current therapeutic strategies follow an algorithm that takes an individualized account of the severity of epistaxis and quality of life [6]. A multistage concept for HHT nose bleeds includes laser therapy and surgery, as well as drug therapies. In addition, screening examinations to determine the effects of the internal organs (mainly lung, liver, brain, and gastro-intestinal tract) are carried out. Visceral lesions which require treatment, should be treated in interdisciplinary cooperation with the corresponding specialized department.

Currently, there is no standardized tool to record treatment success or show the need for the escalation of treatment. With HHT being an orphan disease, patients are aware that the knowledge of non-specialized physicians about HHT is limited. Therefore, information about Rendu-Osler-Weber syndrome, the self-treatment of epistaxis, antibiotic prophylaxis and the necessary organ examinations are crucial for attending physicians and patients. To increase awareness of this rare disease and to document the ongoing treatment by the specialized center, a patient brochure in combination with a treatment documentation tool was developed.

## 2. Materials and Methods

### 2.1. Osler Calendar

Being part of the center of orphan diseases at the Regensburg University Medical Center, the Department of Otorhinolaryngology developed a care plan, called the Osler Calendar, for patients with HHT and their attending physicians. The aim was to provide basic knowledge about the disease such as how to self-manage the symptom of acute epistaxis and when antibiotic prophylaxis is necessary. Additionally, the calendar focused on the need of organ screening examination and its documentation. At last, the calendar documented the patient’s satisfaction and clinical parameters next to the therapy conducted by the specialized center. For a PDF version of the Osler Calendar see Supplementary Materials.

### 2.2. Multistep Treatment Approach

From January 2018 onwards, the treatment of HHT patients at the Department of Otorhinolaryngology at the Regensburg University Medical Center was documented with the Osler Calendar. The first step of the multistep treatment approach was protecting the nasal mucosa by daily application of creams, oils, splints, hygroscopic sprays or temporary nasal occlusion with adhesive plaster [7]. The next step comprised the use of pulsed Nd:YAG laser (infrared 1064 nm) and the TruBlue laser (blue light 445 nm) [5,8]. Coagulation with high-frequency alternating current was used on high-flow shunts. If these measures failed to succeed, surgery was indicated to close feeding vessels or the nidus was resected [1]. In some patients, local endonasal administration of 3.75 mg Bevacizumab per side was conducted in addition to laser therapy or surgery [9]. Patients not eligible for surgical intervention or suffering from blood loss due to visceral lesion, received systemic Bevacizumab treatment according to previously published protocols [10].

### 2.3. Patients

A questionnaire was handed to patients (*n* = 54), who received treatment for HHT at a tertiary referral center specializing in HHT between 2019 and 2020. All patients were diagnosed with HHT clinically according to the Curaçao criteria (all patients were positive for at least three out of four criteria) and used the Osler Calendar for at least one year. Pediatric patients were excluded from this study, as symptoms requiring interventions usually occurred later in life. This retrospective descriptive study was based on an anonymous survey using a questionnaire developed to evaluate the use of the Osler Calendar. The study was conducted at the Department of Otorhinolaryngology, University Medical Center Regensburg, Germany, according to the principles of Helsinki and approved by the Local Ethics Committee (No. 17-854-101). Informed consent was obtained from all subjects involved in the study.

### 2.4. Questionnaire to Review the Osler Calendar from the Patient’s Point of View

Thirteen months after introducing the Osler Calendar to our clinical routine we evaluated current treatment strategies based on the information given by the Osler-Calendar and reviewed the calendar from the patient’s point of view. A questionnaire was developed to determine if patients used the Osler Calendar for information about their disease, self-treatment of epistaxis, antibiotic prophylaxis, and necessary organ screening examinations. Those questions were answered with yes or no. The questions, if organ-screening was important to patients before and after the use of the Osler Calendar and if the Osler Calendar documented the course of the disease adequately, were answered by a five-point likert scale. The questions of whether attending physicians of patients with HHT used the Osler Calendar for information about the disease and the ongoing treatment were answered with yes or no. A three-point likert scale was applied to answer the question of whether the Osler Calendar improved patient’s treatment and a visual analogue scale from zero to ten answered how content patients were regarding the frequency of epistaxis.

### 2.5. Statistics

Data processing and statistical analysis was based on the statistics software, SPSS Statistics 25 (International Business Machines Corporation; Armonk, NY, USA). Microsoft Excel (©2021 Microsoft Corporation) was used for data collection and the displaying of results. Categorical or nominal data were shown in pie charts. The graphs displayed the number of patients in the upper row and the percentage of all patients (*n* = 54) in the lower row. Results of the visual analogue scale were displayed as box plot (medians and interquartile range).

## 3. Osler Calendar Content and Results

### 3.1. Information on HHT and Self-Treatment for Nosebleeds

**Figure 1:**
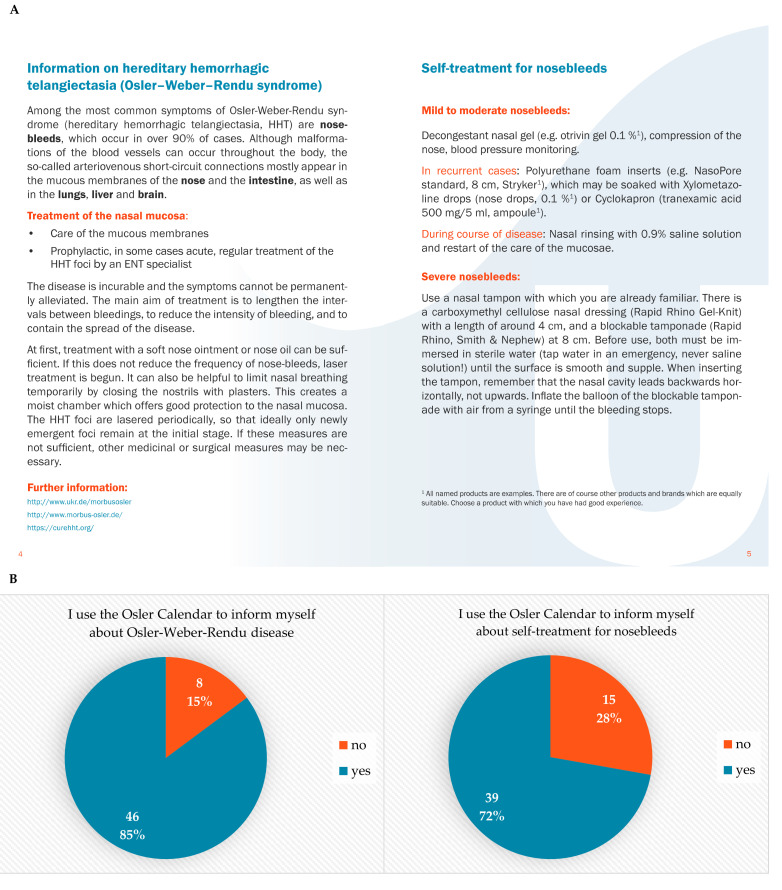
(**A**) Information on HHT and self-treatment for nosebleeds. (**B**) Use of the Osler Calendar for information on HHT and self-treatment of nosebleeds.

The first aim of the Osler Calendar was to inform patients briefly about the most common symptoms of Osler-Weber-Rendu syndrome and how to approach them (Figure 1A). The most common symptom is epistaxis, which occurs in over 90% of cases. Nosebleeds are caused by vascular malformations which appear in the mucous membranes of the nose, but also in the intestine, lungs, liver, and brain. Currently, the disease is incurable, and the symptoms cannot be permanently alleviated. The main aim of treatment is to lengthen the intervals between bleedings, to reduce the intensity of bleeding, to contain the spread of the disease and to prevent the complications resulting from visceral lesions (see Section 3.2). The treatment of the nasal mucosa follows a multi-step approach, where the first step is the prevention of bleeding thorough the care of the mucous membranes by the patient with a soft nose ointment or nose oil. It can also be helpful to limit nasal breathing temporarily by closing the nostrils with plasters (e.g., Micropore^TM^ 2.5 cm or hydrocolloid nasal tape). Thus the mucous membranes are protected from drying out, crusting and microtrauma due to air flow [7,11,12]. These “second-hits” are discussed in the literature for triggering the growth of the lesions [13]. In acute situations, decongestant nasal gel or spray, compression of the nose, and blood pressure monitoring are recommended. For more severe bleedings, nasal inserts (e.g., Stypro^®^ Standard, Curasan; NasoPore^®^ Standard, Stryker, Kalamazoo, MI, USA), which may be soaked with decongestant nose drops or tranexamic acid (500 mg/5 mL), can be prescribed to the patient. For epistaxis, which is not controlled by these measures, carboxymethyl cellulose nasal dressings (e.g., Rapid Rhino^TM^ Gel-Knit, Smith & Nephew) and inflatable nasal tamponades (e.g., Rapid Rhino^TM^, Smith & Nephew) are available. The Osler Calendar provides an overview of the disease pattern of HHT and provides online links where patients can find more detailed information. After one year of using the Osler Calendar, 85% of patients (*n* = 46) claimed to use the calendar to gain information about Osler-Weber-Rendu disease (Figure 1B, left panel). Furthermore, the Osler Calendar informed patients of how to approach nosebleeds of varying severities and provided detailed instructions on nasal packing. Seventy two percent (*n* = 39) of patients stated, that they used the Osler Calendar for instructions about self-treatment for nosebleed (Figure 1B, right panel).

### 3.2. Information on Screening Examinations for Visceral Lesions of HHT

**Figure 2:**
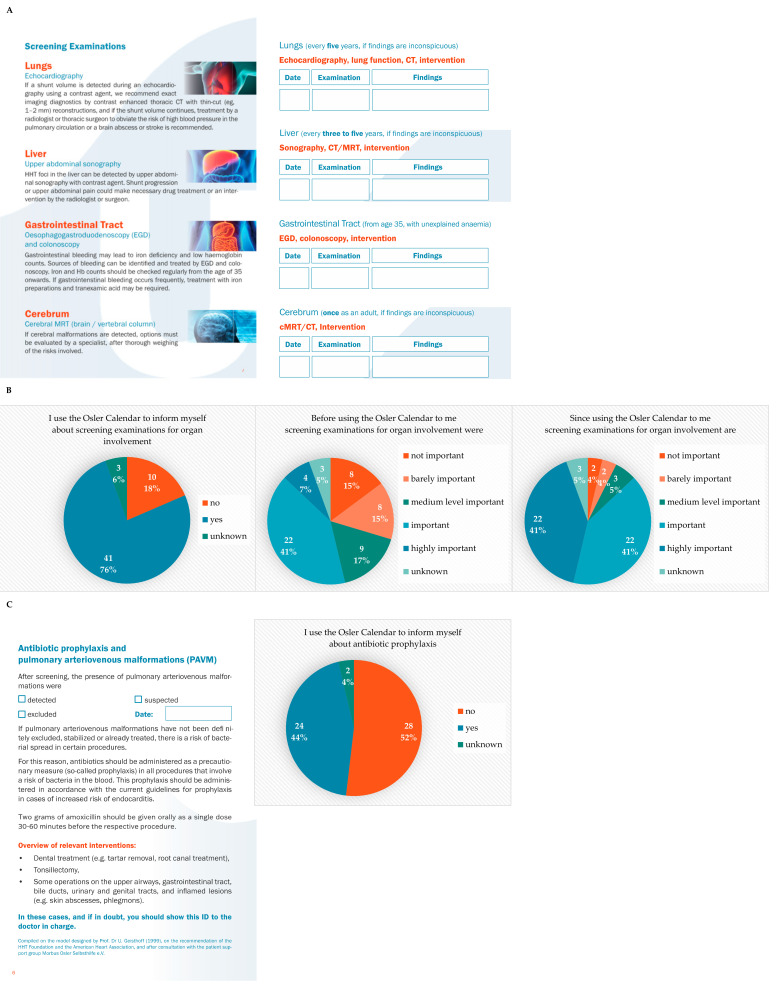
(**A**) Information on screening examinations for visceral lesions of HHT. (**B**) Use of the Osler Calendar for screening examinations for organ involvement. (**C**) Information on antibiotic prophylaxis.

According to the Second International Guidelines for the Diagnosis and Management of HHT, screening examinations for visceral lesions are recommended [2]. All patients with possible or confirmed HHT should be screened for vascular anomalies involving the lungs, liver and the brain. The screening for pulmonary arteriovenous malformations (PAVM) is performed by a transthoracic contrast echocardiography as the initial screening test. In case PAVMs are suspected, the diagnosis is confirmed or dismissed by contrast-enhanced, thoracic, computed tomography (CT) with thin-slice (1 mm) reconstructions. The patients with documented PAVMs are at a higher risk of brain abscess, stroke, and myocardial ischemia due to a paradoxical embolism (septic, air-associated or by blood clots). Therefore, patients should be advised to receive antibiotic prophylaxis for procedures with a risk of bacteremia and to avoid scuba diving. Furthermore, clinicians should provide a long-term follow-up for patients with PAVMs to assess the growth of the untreated lesion or reperfusion of treated AVMs. The screening of liver vascular malformations (VM) is performed by Doppler ultrasound with a contrast enhancement, multiphase contrast CT scan, or contrast abdominal magnetic resonance imaging (MRI). Hepatic VMs are found in 40–70% of HHT patients [14], only patients with symptoms (including heart failure, pulmonary hypertension, abnormal cardiac biomarkers, abnormal liver function tests, abdominal pain, portal hypertension or encephalopathy require medical intervention and should be managed and followed up by a specialized center. MRI is used to screen for brain VMs. If lesions are detected, the individualized management should be carried out in a center with neurovascular expertise. For patients with anemia disproportionate to the severity of epistaxis, esophagogastroduodenoscopy (EGD) is recommended to identify suspected HHT-related bleeding. Patients who meet colorectal cancer screening criteria, and patients with SMAD4 mutations, should undergo colonoscopy. Iron deficiency and low hemoglobin counts can be caused by gastrointestinal bleeding. Therefore, the parameters should be checked regularly from the age of 35. Treatment may require the argon plasma coagulation of the gastrointestinal lesions, iron preparations, intravenous bevacizumab and/or tranexamic acid [2,10,15]. The Osler Calendar provides an overview of the necessary screening examinations, documents their results, and reminds patients of the necessary follow ups (Figure 2A). After one year of using the Osler Calendar, 76% of patients (*n* = 41) stated that they used the calendar to inform themselves about screening examinations for organ involvement in HHT (Figure 2B, left panel). The need for screening examinations was important or highly important to 48% (*n* = 26) of patients (Figure 2B, middle panel). After one year of using the Osler Calendar this number increased to 81% (*n* = 44) (Figure 2B, right panel). Furthermore, the Osler Calendar informs patients about antibiotic prophylaxis for patients with PAVMs. However, 52% (*n* = 28) of patients stated that they did not inform themselves about antibiotic prophylaxis with the Osler Calendar (Figure 2C).

### 3.3. Documentation of Epistaxis Treatment and Patient Outcome

**Figure 3:**
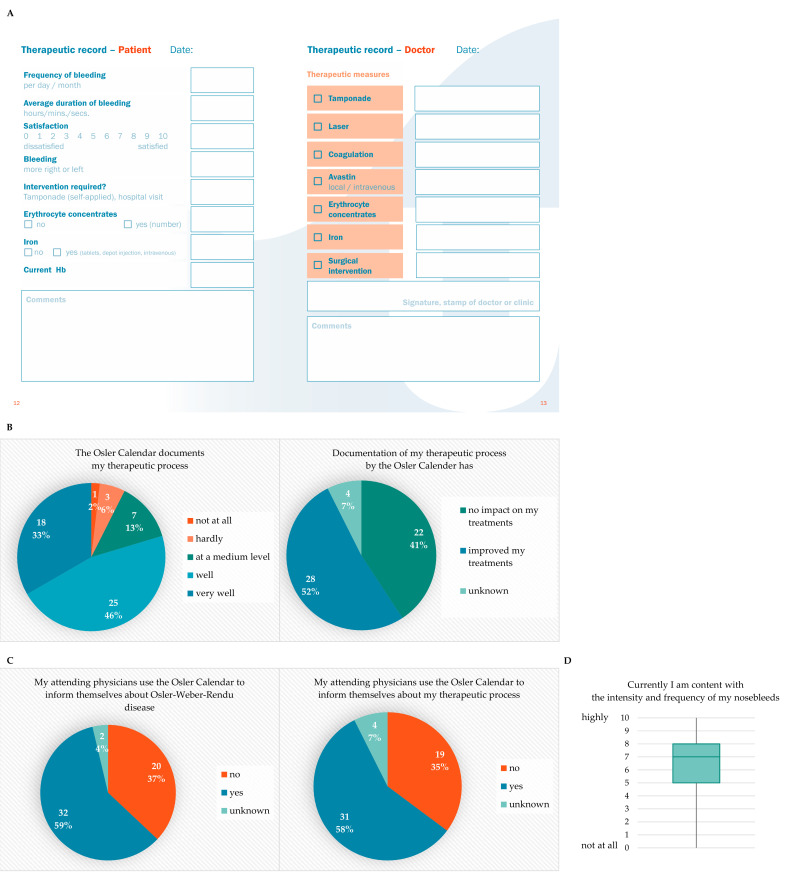
(**A**) Documentation of epistaxis treatment and patient outcome. (**B**) Use of the Osler Calendar for documentation of the therapeutic process. (**C**) Use of the Osler Calendar by attending physicians. (**D**) Patients’ satisfaction with intensity and frequency of nosebleeds.

Epistaxis is the main symptom of HHT. The intensity varies from mild, scarce bleeding to life-threatening epistaxis requiring nasal packing, hospitalization, and frequent blood transfusion, thus limiting the patient’s quality of life [5]. The current therapeutic strategies follow an algorithm that takes an individualized account of the severity of epistaxis and the impairment of quality of life [1,6,16,17]. The first step is the care of the mucous membranes by the patient, as described above. The next steps in epistaxis treatment are carried out by the attending physician. Laser treatments by an otorhinolaryngologist experienced in the treatment of epistaxis in HHT are recommended [8,18,19,20,21]. The HHT foci are lasered periodically so that, ideally, only newly emergent foci remain at the initial stage. In the cases of severe epistaxis, the coagulation of the intranasal lesions can be necessary. These measures can be performed in-office by local anesthesia, but excessive bleeding can require interventions (laser therapy, coagulation and the closure of feeders, e.g., closure of the sphenopalatine artery, or sclerotherapy) under general anesthesia [16,20,22]. If those measures cannot improve the severity of epistaxis or if the involvement of other organs requires treatment, such as the gastrointestinal tract or the liver, drug therapies (tranexamic acid, bevacizumab, thalidomide and tacrolimus) are available [10,15,23,24,25]. The Osler Calendar documents the therapeutic measures since the last patient contact, including iron supplements, blood transfusions, nasal packing, laser therapy, coagulation, surgical interventions, and drug treatments (Figure 3A, right). As well as the therapeutic measures by the attending physician, the Osler Calendar reports the frequency of the bleeding, the average duration of the bleeding, the predominant side of the nose bleeds, the satisfaction with the current bleeding situation, the necessary interventions by the patient (nasal packing, hospitalization and previous treatments of anemia such as blood transfusions or iron supplements) and the current hemoglobin levels (Figure 3A left). The information is documented repetitively at every in-office appointment, as well as at every hospital stay. Therefore, the treatment success measured by the Epistaxis Severity Score, as well as patient satisfaction, can be traced over the course of time and treatment and can be adapted accordingly. After one year of use, 79% (*n* = 43) of patients stated that the Osler Calendar documented their therapeutic process either well or very well (Figure 3B, left panel) and 52% (*n* = 28) saw an improvement in their therapeutic process due to the documentation by the Osler Calendar (Figure 3B, right panel). Furthermore, the Osler Calendar is a tool used to inform healthcare providers about the ongoing treatment of the patient. In our study, 59% (*n* = 32) of the patients documented that their treating physicians used the Osler Calendar to inform themselves about Osler-Weber-Rendu disease (Figure 3C, left panel) and 58% (*n* = 31) of the patients reported that the Osler Calendar was used by their attending physicians for information about the therapeutic process of epistaxis treatment (Figure 3C, right panel). The overall satisfaction of the patients with the intensity and frequency of the nosebleeds in our collective of 54 patients showed a median of seven (interquartile range, IQR 5–8) on a visual analogue scale (VAS) from zero (not content at all) to ten (highly content) (Figure 3D).

## 4. Discussion

In cancer treatment survivorship, care plans by the American Society of Clinical Oncology, consisting of treatment summaries and follow-up care plans, are well established essential components of patient care. These care plans enhance the communication between the medical team and the patient, as well as communication and coordination of care between the attending physicians and the primary care providers [26]. Following the concept of delivering patient-centered care, we developed a HHT patient care plan called the Osler Calendar to improve the communication with patients and primary care providers. Furthermore, the calendar intended to help physicians working with patients with this rare disease to recognize the importance of screening examinations and patient referral to specialized centers.

After one year of using the Osler Calendar, 85% of patients confirmed using the calendar to gain information about Osler-Weber-Rendu disease. However, only 59% of the patients documented that their primary care providers used the Osler Calendar to inform themselves about the disease and 58% about the therapeutic process of the epistaxis. The reasons for the less frequent use of the Osler Calendar by the primary care providers could be shortage of time during appointments or the missing transfer of the Osler Calendar between the patient and the primary care provider [27]. To overcome this obstacle, an electronical version of the Osler Calendar would be helpful. An app for patient education about HHT and tracking health data was previously developed [28]. In addition to information about HHT and health tracking executed by the patient, the Osler Calendar summarizes notes by the attending physician and tracks the treatment success. Therefore, the dynamic of the disease is documented and can be used to measure the effectiveness of the current therapeutic strategy. Seventy-nine percent of patients confirmed that the Osler Calendar documented their therapeutic process either well or very well and 52% even saw an improvement in their therapeutic process due to the documentation by the Osler Calendar. Similar to this result, it was reported that health tracking had a positive impact on health-related behaviors, adherence to medication or therapy, and the knowledge enhancement related to clinical procedures [29]. In the future, an app version of the calendar for iOS and Android will further improve communication with patients and primary care providers.

Next to improvement of communication, an additional objective of the Osler Calendar is to increase knowledge about HHT, treatment options, the necessary screening examinations, and the possible need for antibiotic prophylaxis. 72% of patients used the Osler Calendar for instructions about self-treatment for nosebleeds, 76% for information about screening examinations for organ involvement. The use of the Osler Calendar increased patients understanding for the need of organ screening from 48% to 81% of patients. The importance of this result is underlined by a recent study showing that the life expectancy of HHT patients systematically screened for HHT-related organ involvement is similar compared to the non-HHT control group [30]. However, only 44% of patients stated that they do inform themselves about antibiotic prophylaxis with the Osler Calendar. This number is concordant with 50% of HHT patients overall being affected by PAVMs [31]. Detailed information about the need for antibiotic prophylaxis for patients with diagnosed PAVM by the attending physician as well as patient training for nasal packing is needed to ensure correct use and patient safety [32,33].

To our knowledge, this is the first study documenting the effect of a tracking calendar for HHT used by patients and clinicians alike. The limitations of this study are the retrospective character of the analyses and the survey of patients with a non-validated questionnaire. The patient satisfaction with the ongoing treatment could be seen as biased due to the monocentric approach of the study. The study was performed at a tertiary center specialized in the treatment of HHT. The overall patient satisfaction with the intensity and frequency of the nosebleeds in the study collective of 54 patients showed a median of seven on a visual analogue scale, which was equal to the results seen in the other specialized centers using the same treatment modalities [8]. In the future, an app-based Osler Calendar used and validated by several specialized tertiary centers is needed to improve patient outcome.

## 5. Conclusions

In summary a patient care plan used by specialized centers, primary care providers, and the patient can provide information about the rare disease of HHT, necessary organ screening examinations, and current treatment strategies of the disease. By merging patient information and treatment plans, the optimal care for each individual patient can be achieved and patient outcome and quality of life can be improved.

## Supplementary Materials

The following are available online at https://www.mdpi.com/article/10.3390/jcm10204720/s1, PDF version of the Osler Calendar, written by Caroline Seebauer, designed by Referat UK1 Regensburg University Medical Center, Regensburg, Germany. Pictures on page one and seven by adimas/Fotolia, pictures on page seven by yodiyim, Naeblys, nerthuz/Fotolia. Current version June 2019. English and German versions are available. Use, distribution, publication, replication, and demonstration of the Osler Calendar is only permitted after author’s consent. For requesting permission to reproduce, reprint or translate the Osler Calendar material please contact Caroline T. Seebauer, Department of Otolaryngology, University Medical Center Regensburg, Franz-Josef-Strauß-Allee 11, 93053 Regensburg, Germany, Phone +49 941 944 9410, Email caroline.seebauer@ukr.de.

## Abbreviations

AVM: arteriovenous malformations; CVM: cerebral vascular malformation; EGD: esophagogastroduodenoscopy; HHT: hereditary hemorrhagic telangiectasia; MRI: magnetic resonance imaging; PAVM: pulmonary arteriovenous malformations; CT: computed tomography; VM: vascular malformations.
