# Supplementary material for: Hereditary Hemorrhagic Telangiectasia: Success of the Osler Calendar for Documentation of Treatment and Course of Disease
Source: J Clin Med. 2021 Oct 14;10(20):4720. doi: 10.3390/jcm10204720 (PMC8541180; doi:10.3390/jcm10204720)
Supplement: Supplementary file 1 [file jcm-10-04720-s001.zip › jcm-1383909-supplementary.pdf]

SPITZE IN DER MEDIZIN. MENSCHLICH IN DER BEGEGNUNG.

UKR Universitätsklinikum Regensburg

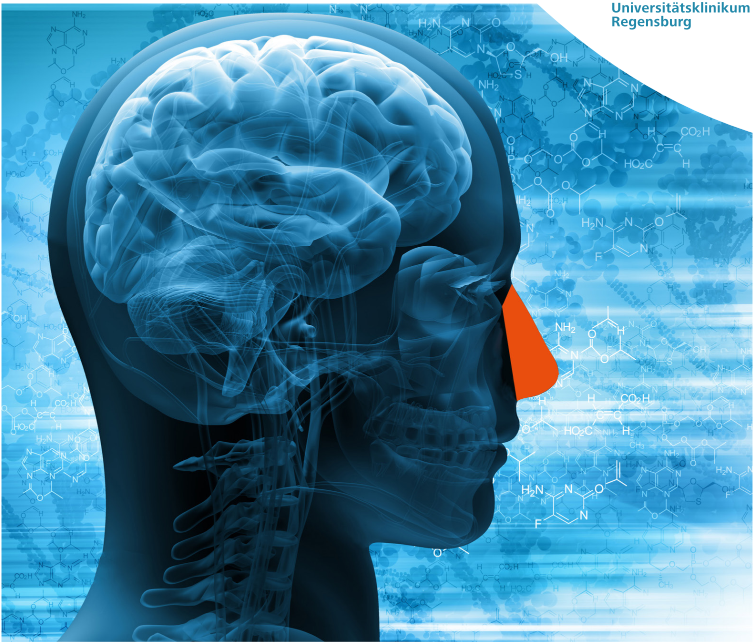

OSLER–WEBER–RENDU SYNDROME
Calendar

Please keep in a safe place and bring to every consultation!

Department of Otorhinolaryngology, University Hospital Regensburg, Germany

ukr.de

DIE DEUTSCHEN UNIVERSITÄTSKLINIKA

Emergency ID 3

Information on hereditary hemorrhagic telangiectasia (Osler–Weber–Rendu syndrome) 4

Self-treatment for nosebleeds 5

Antibiotic prophylaxis and pulmonary arteriovenous malformations (PAVM) 6

Screening examinations … 7
- … Lungs 8
- … Liver 9
- … Gastrointestinal tract 10
- … Cerebrum 11

Therapeutic process 12

Notes 38

Issued on/by

Date, stamp of doctor or clinic

Emergency ID

Surname, forename

Date of birth

Street

Postcode, town

Telephone

Allergies (e.g. medications, others)

List of medications (Date: ______________)

Information on hereditary hemorrhagic telangiectasia (Osler–Weber–Rendu syndrome)

Among the most common symptoms of Osler-Weber-Rendu syndrome (hereditary hemorrhagic telangiectasia, HHT) are **nose-bleeds**, which occur in over 90% of cases. Although malformations of the blood vessels can occur throughout the body, the so-called arteriovenous short-circuit connections mostly appear in the mucous membranes of the **nose** and the **intestine**, as well as in the **lungs**, **liver** and **brain**.

Treatment of the nasal mucosa:

- Care of the mucous membranes
- Prophylactic, in some cases acute, regular treatment of the HHT foci by an ENT specialist

The disease is incurable and the symptoms cannot be permanently alleviated. The main aim of treatment is to lengthen the intervals between bleedings, to reduce the intensity of bleeding, and to contain the spread of the disease.

At first, treatment with a soft nose ointment or nose oil can be sufficient. If this does not reduce the frequency of nose-bleeds, laser treatment is begun. It can also be helpful to limit nasal breathing temporarily by closing the nostrils with plasters. This creates a moist chamber which offers good protection to the nasal mucosa. The HHT foci are lasered periodically, so that ideally only newly emergent foci remain at the initial stage. If these measures are not sufficient, other medicinal or surgical measures may be necessary.

Further information:

http://www.ukr.de/morbusosler

http://www.morbus-osler.de/

https://curehht.org/

Self-treatment for nosebleeds

Mild to moderate nosebleeds:

Decongestant nasal gel (e.g. otrivin gel 0.1 %[1]), compression of the nose, blood pressure monitoring.

In recurrent cases: Polyurethane foam inserts (e.g. NasoPore standard, 8 cm, Stryker[1]), which may be soaked with Xylometazoline drops (nose drops, 0.1 %[1]) or Cyclokapron (tranexamic acid 500 mg/5 ml, ampoule[1]).

During course of disease: Nasal rinsing with 0.9% saline solution and restart of the care of the mucosae.

Severe nosebleeds:

Use a nasal tampon with which you are already familiar. There is a carboxymethyl cellulose nasal dressing (Rapid Rhino Gel-Knit) with a length of around 4 cm, and a blockable tamponade (Rapid Rhino, Smith & Nephew) at 8 cm. Before use, both must be immersed in sterile water (tap water in an emergency, never saline solution!) until the surface is smooth and supple. When inserting the tampon, remember that the nasal cavity leads backwards horizontally, not upwards. Inflate the balloon of the blockable tamponade with air from a syringe until the bleeding stops.

[1] All named products are examples. There are of course other products and brands which are equally suitable. Choose a product with which you have had good experience.

Antibiotic prophylaxis and pulmonary arteriovenous malformations (PAVM)

After screening, the presence of pulmonary arteriovenous malformations were

☐ detected ☐ suspected

☐ excluded **Date:** ____________

If pulmonary arteriovenous malformations have not been defi nitely excluded, stabilized or already treated, there is a risk of bacterial spread in certain procedures.

For this reason, antibiotics should be administered as a precautionary measure (so-called prophylaxis) in all procedures that involve a risk of bacteria in the blood. This prophylaxis should be administered in accordance with the current guidelines for prophylaxis in cases of increased risk of endocarditis.

Two grams of amoxicillin should be given orally as a single dose 30-60 minutes before the respective procedure.

Overview of relevant interventions:

- Dental treatment (e.g. tartar removal, root canal treatment),
- Tonsillectomy,
- Some operations on the upper airways, gastrointestinal tract, bile ducts, urinary and genital tracts, and inflamed lesions (e.g. skin abscesses, phlegmons).

In these cases, and if in doubt, you should show this ID to the doctor in charge.

Compiled on the model designed by Prof. Dr U. Geisthoff (1999), on the recommendation of the HHT Foundation and the American Heart Association, and after consultation with the patient support group Morbus Osler Selbsthilfe e.V.

Screening Examinations

Lungs

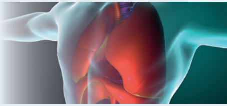

Echocardiography

If a shunt volume is detected during an echocardiography using a contrast agent, we recommend exact imaging diagnostics by contrast enhanced thoracic CT with thin-cut (eg, 1–2 mm) reconstructions, and if the shunt volume continues, treatment by a radiologist or thoracic surgeon to obviate the risk of high blood pressure in the pulmonary circulation or a brain abscess or stroke is recommended.

Liver

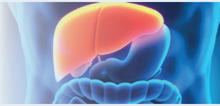

Upper abdominal sonography

HHT foci in the liver can be detected by upper abdominal sonography with contrast agent. Shunt progression or upper abdominal pain could make necessary drug treatment or an intervention by the radiologist or surgeon.

Gastrointestinal Tract

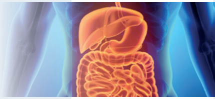

Oesophagogastroduodenoscopy (EGD) and colonoscopy

Gastrointestinal bleeding may lead to iron deficiency and low haemoglobin counts. Sources of bleeding can be identified and treated by EGD and colonoscopy. Iron and Hb counts should be checked regularly from the age of 35 onwards. If gastrointenstinal bleeding occurs frequently, treatment with iron preparations and tranexamic acid may be required.

Cerebrum

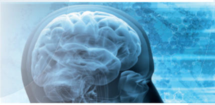

Cerebral MRT (brain / vertebral column)

If cerebral malformations are detected, options must be evaluated by a specialist, after thorough weighing of the risks involved.

Screening Examinations

Lungs (every **five** years, if findings are inconspicuous)

Echocardiography, lung function, CT, intervention

Date	Examination	Findings

Screening Examinations

Liver (every **three to five** years, if findings are inconspicuous)

Sonography, CT/MRT, intervention

Date	Examination	Findings

Screening Examinations

Gastrointestinal Tract (from age 35, with unexplained anaemia)

EGD, colonoscopy, intervention

Date	Examination	Findings

Screening Examinations

Cerebrum (**once** as an adult, if findings are inconspicuous)

cMRT/CT, Intervention

Date	Examination	Findings

Notes

Therapeutic record – Patient Date:

Frequency of bleeding
per day / month

Average duration of bleeding
hours/mins./secs.

Satisfaction
0 1 2 3 4 5 6 7 8 9 10
dissatisfied satisfied

Bleeding
more right or left

Intervention required?
Tamponade (self-applied), hospital visit

Erythrocyte concentrates
☐ no ☐ yes (number)

Iron
☐ no ☐ yes (tablets, depot injection, intravenous)

Current Hb

Comments

Therapeutic record – Doctor Date:

Therapeutic measures

☐ **Tamponade**	
☐ **Laser**	
☐ **Coagulation**	
☐ **Avastin** local / intravenous	
☐ **Erythrocyte concentrates**	
☐ **Iron**	
☐ **Surgical intervention**	

Signature, stamp of doctor or clinic

Comments

Therapeutic record – Patient Date:

Frequency of bleeding
per day / month

Average duration of bleeding
hours/mins./secs.

Satisfaction
0 1 2 3 4 5 6 7 8 9 10
dissatisfied satisfied

Bleeding
more right or left

Intervention required?
Tamponade (self-applied), hospital visit

Erythrocyte concentrates
☐ no ☐ yes (number)

Iron
☐ no ☐ yes (tablets, depot injection, intravenous)

Current Hb

Comments

Therapeutic record – Doctor Date:

Therapeutic measures

☐ **Tamponade**	
☐ **Laser**	
☐ **Coagulation**	
☐ **Avastin** local / intravenous	
☐ **Erythrocyte concentrates**	
☐ **Iron**	
☐ **Surgical intervention**	

Signature, stamp of doctor or clinic

Comments

Therapeutic record – Patient   Date:

**Frequency of bleeding**<br>per day / month	
**Average duration of bleeding**<br>hours/mins./secs.	
**Satisfaction**<br>0 1 2 3 4 5 6 7 8 9 10<br>dissatisfied satisfied	
**Bleeding**<br>more right or left	
**Intervention required?**<br>Tamponade (self-applied), hospital visit	
**Erythrocyte concentrates**<br>☐ no ☐ yes (number)	
**Iron**<br>☐ no ☐ yes (tablets, depot injection, intravenous)	
**Current Hb**	

Comments

Therapeutic record – Doctor Date:

Therapeutic measures

☐ **Tamponade**	
☐ **Laser**	
☐ **Coagulation**	
☐ **Avastin** local / intravenous	
☐ **Erythrocyte concentrates**	
☐ **Iron**	
☐ **Surgical intervention**	

Signature, stamp of doctor or clinic

Comments

Therapeutic record – Patient   Date:

Frequency of bleeding
per day / month

Average duration of bleeding
hours/mins./secs.

Satisfaction
0 1 2 3 4 5 6 7 8 9 10
dissatisfied satisfied

Bleeding
more right or left

Intervention required?
Tamponade (self-applied), hospital visit

Erythrocyte concentrates
☐ no ☐ yes (number)

Iron
☐ no ☐ yes (tablets, depot injection, intravenous)

Current Hb

Comments

Therapeutic record – Doctor   Date:

Therapeutic measures

☐ **Tamponade**	
☐ **Laser**	
☐ **Coagulation**	
☐ **Avastin** local / intravenous	
☐ **Erythrocyte concentrates**	
☐ **Iron**	
☐ **Surgical intervention**	

Signature, stamp of doctor or clinic

Comments

Therapeutic record – Patient Date:

Frequency of bleeding
per day / month

Average duration of bleeding
hours/mins./secs.

Satisfaction
0 1 2 3 4 5 6 7 8 9 10
dissatisfied satisfied

Bleeding
more right or left

Intervention required?
Tamponade (self-applied), hospital visit

Erythrocyte concentrates
☐ no ☐ yes (number)

Iron
☐ no ☐ yes (tablets, depot injection, intravenous)

Current Hb

Comments

Therapeutic record – Doctor   Date:

Therapeutic measures

☐ **Tamponade**	
☐ **Laser**	
☐ **Coagulation**	
☐ **Avastin** local / intravenous	
☐ **Erythrocyte concentrates**	
☐ **Iron**	
☐ **Surgical intervention**	

Signature, stamp of doctor or clinic

Comments

Therapeutic record – Patient   Date:

Frequency of bleeding
per day / month

Average duration of bleeding
hours/mins./secs.

Satisfaction
0 1 2 3 4 5 6 7 8 9 10
dissatisfied satisfied

Bleeding
more right or left

Intervention required?
Tamponade (self-applied), hospital visit

Erythrocyte concentrates
☐ no ☐ yes (number)

Iron
☐ no ☐ yes (tablets, depot injection, intravenous)

Current Hb

Comments

Therapeutic record – Doctor   Date:

Therapeutic measures

☐ **Tamponade**	
☐ **Laser**	
☐ **Coagulation**	
☐ **Avastin** local / intravenous	
☐ **Erythrocyte concentrates**	
☐ **Iron**	
☐ **Surgical intervention**	

Signature, stamp of doctor or clinic

Comments

Therapeutic record – Patient   Date:

Frequency of bleeding
per day / month

Average duration of bleeding
hours/mins./secs.

Satisfaction
0 1 2 3 4 5 6 7 8 9 10
dissatisfied satisfied

Bleeding
more right or left

Intervention required?
Tamponade (self-applied), hospital visit

Erythrocyte concentrates
☐ no ☐ yes (number)

Iron
☐ no ☐ yes (tablets, depot injection, intravenous)

Current Hb

Comments

Therapeutic record – Doctor Date:

Therapeutic measures

☐ **Tamponade**	
☐ **Laser**	
☐ **Coagulation**	
☐ **Avastin** local / intravenous	
☐ **Erythrocyte concentrates**	
☐ **Iron**	
☐ **Surgical intervention**	

Signature, stamp of doctor or clinic

Comments

Therapeutic record – Patient Date:

Frequency of bleeding
per day / month

Average duration of bleeding
hours/mins./secs.

Satisfaction
0 1 2 3 4 5 6 7 8 9 10
dissatisfied satisfied

Bleeding
more right or left

Intervention required?
Tamponade (self-applied), hospital visit

Erythrocyte concentrates
☐ no ☐ yes (number)

Iron
☐ no ☐ yes (tablets, depot injection, intravenous)

Current Hb

Comments

Therapeutic record – Doctor   Date:

Therapeutic measures

☐ **Tamponade**	
☐ **Laser**	
☐ **Coagulation**	
☐ **Avastin** local / intravenous	
☐ **Erythrocyte concentrates**	
☐ **Iron**	
☐ **Surgical intervention**	

Signature, stamp of doctor or clinic

Comments

Therapeutic record – Patient Date:

**Frequency of bleeding**<br>per day / month	
**Average duration of bleeding**<br>hours/mins./secs.	
**Satisfaction**<br>0 1 2 3 4 5 6 7 8 9 10<br>dissatisfied satisfied	
**Bleeding**<br>more right or left	
**Intervention required?**<br>Tamponade (self-applied), hospital visit	
**Erythrocyte concentrates**<br>☐ no ☐ yes (number)	
**Iron**<br>☐ no ☐ yes (tablets, depot injection, intravenous)	
**Current Hb**	

Comments

Therapeutic record – Doctor   Date:

Therapeutic measures

☐ **Tamponade**	
☐ **Laser**	
☐ **Coagulation**	
☐ **Avastin** local / intravenous	
☐ **Erythrocyte concentrates**	
☐ **Iron**	
☐ **Surgical intervention**	

Signature, stamp of doctor or clinic

Comments

Therapeutic record – Patient Date:

Frequency of bleeding
per day / month

Average duration of bleeding
hours/mins./secs.

Satisfaction
0 1 2 3 4 5 6 7 8 9 10
dissatisfied satisfied

Bleeding
more right or left

Intervention required?
Tamponade (self-applied), hospital visit

Erythrocyte concentrates
☐ no ☐ yes (number)

Iron
☐ no ☐ yes (tablets, depot injection, intravenous)

Current Hb

Comments

Therapeutic record – Doctor   Date:

Therapeutic measures

☐ **Tamponade**	
☐ **Laser**	
☐ **Coagulation**	
☐ **Avastin** local / intravenous	
☐ **Erythrocyte concentrates**	
☐ **Iron**	
☐ **Surgical intervention**	

Signature, stamp of doctor or clinic

Comments

Therapeutic record – Patient   Date:

Frequency of bleeding
per day / month

Average duration of bleeding
hours/mins./secs.

Satisfaction
0 1 2 3 4 5 6 7 8 9 10
dissatisfied satisfied

Bleeding
more right or left

Intervention required?
Tamponade (self-applied), hospital visit

Erythrocyte concentrates
☐ no ☐ yes (number)

Iron
☐ no ☐ yes (tablets, depot injection, intravenous)

Current Hb

Comments

Therapeutic record – Doctor   Date:

Therapeutic measures

☐ **Tamponade**	
☐ **Laser**	
☐ **Coagulation**	
☐ **Avastin** local / intravenous	
☐ **Erythrocyte concentrates**	
☐ **Iron**	
☐ **Surgical intervention**	

Signature, stamp of doctor or clinic

Comments

Therapeutic record – Patient   Date:

Frequency of bleeding
per day / month

Average duration of bleeding
hours/mins./secs.

Satisfaction
0 1 2 3 4 5 6 7 8 9 10
dissatisfied satisfied

Bleeding
more right or left

Intervention required?
Tamponade (self-applied), hospital visit

Erythrocyte concentrates
☐ no ☐ yes (number)

Iron
☐ no ☐ yes (tablets, depot injection, intravenous)

Current Hb

Comments

Therapeutic record – Doctor Date:

Therapeutic measures

☐ **Tamponade**	
☐ **Laser**	
☐ **Coagulation**	
☐ **Avastin** local / intravenous	
☐ **Erythrocyte concentrates**	
☐ **Iron**	
☐ **Surgical intervention**	

Signature, stamp of doctor or clinic

Comments

Therapeutic record – Patient   Date:

Frequency of bleeding
per day / month

Average duration of bleeding
hours/mins./secs.

Satisfaction
0 1 2 3 4 5 6 7 8 9 10
dissatisfied satisfied

Bleeding
more right or left

Intervention required?
Tamponade (self-applied), hospital visit

Erythrocyte concentrates
☐ no ☐ yes (number)

Iron
☐ no ☐ yes (tablets, depot injection, intravenous)

Current Hb

Comments

Therapeutic record – Doctor   Date:

Therapeutic measures

☐ **Tamponade**	
☐ **Laser**	
☐ **Coagulation**	
☐ **Avastin** local / intravenous	
☐ **Erythrocyte concentrates**	
☐ **Iron**	
☐ **Surgical intervention**	

Signature, stamp of doctor or clinic

Comments

Notes

Notes

Contact:

University Hospital Regensburg
Department of Otorhinolaryngology
Director: Professor Dr. Christopher Bohr
Franz-Josef-Strauß-Allee 11
D-93053 Regensburg
Germany
T: +49 941 944-9410
F: +49 941 944-9402

Text: Dr. Caroline Seebauer
Design: Referat UK1, UKR

Photos:
P.1&7: adimas/Fotolia
P.7: yodiyim, Naeblys, nerthuz/Fotolia

Version: June 2019

www.ukr.de/hno
